# Electrokinetic Analysis of Energy Harvest from Natural Salt Gradients in Nanochannels

**DOI:** 10.1038/s41598-017-13336-w

**Published:** 2017-10-13

**Authors:** Yuhui He, Zhuo Huang, Bowei Chen, Makusu Tsutsui, Xiang Shui Miao, Masateru Taniguchi

**Affiliations:** 1 School of Optical and Electronic Information, Huazhong University of Science and Technology, LuoYu Road, Wuhan, 430074 China; 2 The Institute of Scientific and Industrial Research, Osaka University, 8-1 Mihogaoka, Ibaraki, Osaka, 567-0047 Japan

## Abstract

The Gibbs free energy released during the mixing of river and sea water has been illustrated as a promising source of clean and renewable energy. Reverse electrodialysis (RED) is one major strategy to gain electrical power from this natural salinity, and recently by utilizing nanochannels a novel mode of this approach has shown improved power density and energy converting efficiency. In this work, we carry out an electrokinetic analysis of the work extracted from RED in the nanochannels. First, we outline the exclusion potential effect induced by the inhomogeneous distribution of extra-counterions along the channel axis. This effect is unique in nanochannel RED and how to optimize it for energy harvesting is the central topic of this work. We then discuss two important indexes of performance, which are the output power density and the energy converting efficiency, and their dependence on the nanochannel parameters such as channel material and geometry. In order to yield maximized output electrical power, we propose a device design by stepwise usage of the saline bias, and the lengths of the nanochannels are optimized to achieve the best trade-off between the input thermal power and the energy converting efficiency.

## Introduction

**Figure 1:**
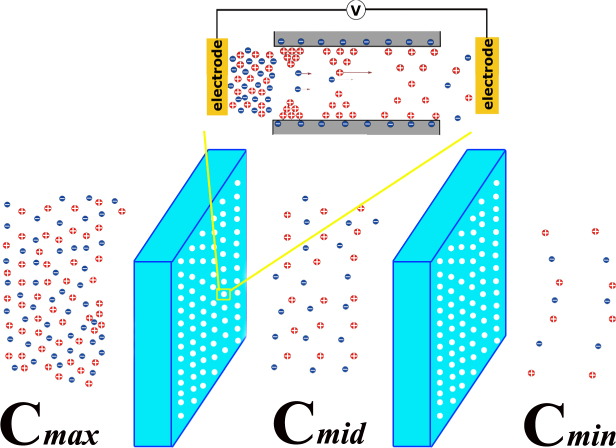
Schematic view of the energy harvesting through sea and river mixing within nanochannels. Inset demonstrates the variation of ion selectivity in saline-concentration-biased nanochannel systems: The high saline concentration ${C}_{max}$ in the left chamber results in densely piling-up of counterions at the left end, while the low ${C}_{min}$ in the right leads to diluter ions at the other end.

The need for clean and sustainable energy sources has boosted a broad spectrum of research interests in the past decades. One potential is the blue energy^1^, which converts the saline gradient power into electricity when fresh water streams flow into the sea as seen in Fig. 1. It has been estimated that nearly 2 terawatt electric power would be potentially harnessed during this process, given the enormous amount of 37,000 km^3^ water released annually from global rivers into the sea^2^. Such an alluring source of renewable and environmentally benign energy has attracted increasing attention, and several strategies have been explored to extract the energy^2–4^, including pressure-retarded osmosis^5–7^, reversed electrodialysis (RED)^1,2,8^, and a few less developed technologies^9–11^. RED was realized by alternately stacking layers of cation/anion selective membranes which separate the diluted and concentrated solutions. The saline concentration difference then drives cations and anions towards opposite directions and consequently an ion current is produced. Here the key technique is the ion-selective property of the membranes which are used for exchanging water and ions between two segregated solutions. Several efforts have been reported on designing and developing the membranes for viable energy harvesting^12,13^. Yet, low energy converting efficiency, low output power density and membrane fouling problems have been the major hurdles on the road towards the practicability of the technology. It is attributed to the lack of cost-effective ion exchange membranes with low ionic resistance and high perm-selectivity for high energy generation rate^2,14^.

The nanochannels, due to the presence of surface charges on the channel walls and the nanometer-scale channel radii, are also capable of being cation- or anion-selective^15–17^. The nanochannel-based energy harvest devices have thus been proposed, and the energy converting in salt-concentration-biased nanochannels can be classified as a specific form of RED^17^. Experimentally, SiO_2_, Al_2_O_3_ nanochannels, polyimide, boron nitride nanotubes *et al*. have been used as nanochannels, and current-voltage characteristic stimulated by saline concentration bias has been reported^17–22^. Very recently by using nanopores drilled in atomic MoS_2_ layer, Feng *et al*. show that a unprecedentedly large electrical currents could be gained^23^. It prompts a prospect of harvesting power density as large as 10^6^ watts per square metre. Theoretical studies have also been performed based on an electrokinetic description of the nanochannel systems, and several numerical results have shown agreement with the experimental observations^2,24,25^.

Despite the above progresses, some fundamental questions concerning the physical mechanisms of RED within nanochnanels still remain. For example, Kim *et al*. observed a ${\rm{\Lambda }}$-shape variation of the induced electrical potential ${\rm{\Delta }}V$ with increasing KCl concentration bias ${C}_{max}/{C}_{min}$ ^3^. The discovery broke a naive expectation of monotonous increasing ${\rm{\Delta }}V$ with ${C}_{max}/{C}_{min}$, and moreover, it implies that a straightforward usage of the large saline difference between the sea and river reservoirs (NaCl with ${C}_{max}/{C}_{min}\,\approx$ 600 mM/10 mM) cannot yield the maximum output power density or efficiency. Hence it calls for theoretical elucidating of the physical mechanisms within the nanochannel RED, as we are going to illustrate. We further investigate two most important indexes of performance, which are the energy conversion efficiency and the output power density, and their dependence on the nanochannel parameters. Based on the clarified physical picture we then propose new strategies to fully utilize the thermal energy released during the sea and river water mixing in nanochannels.

### Physical mechanism of RED in nanochannels

#### Exclusion potential effect

The working principle of RED in nanochannel system is schematically illustrated with negatively charged nanochannel wall (SiO_2_) as an example in the inset of Fig. 1. A salt concentration bias is imposed at the two ends of a fluid channel which is of nanoscale radius. In order to screen the surface charges on the channel wall, a layer of counterions is induced adjacent to the wall, and the thickness of these electrical double layers (EDL) depends on the local concentration of the imposed electrolyte. The larger the salt concentration, the stronger the capability of shielding wall surface charges and thus the thinner the EDL. Hence rather than a layer with invariant thickness along the channel axial direction, the EDL become thicker and thicker from the high concentration end (${C}_{max}$) to the low one (${C}_{min}$). In the open-circuit state, the diffusion of those extra cations along the channel axial direction would result in an electrical potential ${\rm{\Delta }}{V}_{\sigma }$. Besides, the different motilities between Na^+^ and Cl^-^ would also raise a potential ${\rm{\Delta }}{V}_{D}$. In the figure, we point out that ${\rm{\Delta }}{V}_{\sigma }$ can be viewed as a *skin effect* since it is caused by the motion of those counterions within EDL. On the other hand, ${\rm{\Delta }}{V}_{D}$ is a *bulk effect* since it is triggered by the mobility difference between cations and anions in the bulk region. The fact that the thickness of EDL is in the scale of nanometers indicates ${\rm{\Delta }}{V}_{\sigma }$ is a unique effect in nanochannels while ${\rm{\Delta }}{V}_{D}$ is universal in various types of fluid channels. Thereby, this work focuses on understanding ${\rm{\Delta }}{V}_{\sigma }$ induced phenomena and exploring approaches to maximize this exclusion effect for RED based energy converting in nanochannels.

The above physical pictures can be quantitatively demonstrated with the space-charge model^26–28^ or by Teorell-Meyer-Sievers (TMS) model^29,30^. In both theoretical frameworks, the electrical potential $V(r,z)$ inside the channel is divided into electromotive and electrostatic components:

1 $$V(r,z)={V}_{0}(z)+\varphi (r,z)$$

The latter term $\varphi (r,z)$ accounts for the electrostatic effects by those surface charges on the channel wall. In the space-charge model, this term is evaluated via Poisson-Boltzmann equation and it reads as follows for monovalent ions like NaCl in a cylindrical nanochannel:

2 $$\{\begin{array}{rcl}\frac{1}{r}\frac{\partial }{\partial r}(r\frac{\partial \bar{\varphi }(r)}{\partial r}) & = & -\frac{{e}^{2}({C}_{+}-{C}_{-})}{kT\varepsilon }=\frac{\sinh \,\bar{\varphi }}{{\lambda }_{D}^{2}}\\ \frac{\partial \bar{\phi }}{\partial r}{|}_{r\mathrm{=0}} & = & 0\\ \frac{\partial \overline{\varphi }}{\partial r}{|}_{r=R} & = & \frac{e{\sigma }_{w}}{kT\varepsilon }\end{array}$$

In the above $\bar{\varphi }=e\varphi /{k}_{B}T$ is the nondimensionalized electrostatic potential, ${C}_{\pm }$ is the concentration of monovalent cations/anions and it is evaluated through Boltzmann distribution as ${C}_{\pm }={C}_{0}\exp (\pm \bar{\varphi })$, ${\sigma }_{w}$ is the density of surface charges on the channel wall, and ${\lambda }_{D}=\sqrt{\varepsilon {k}_{B}T/2{C}_{0}{e}^{2}}$ is the Debye length which characterizes the thickness of EDL. In our approach the salt concentration along the channel axis, *C*_0_(*z*), is estimated by taking into account the access resistance of the cylindrical nanochannel/nanopore system:^31,32^

3 $${C}_{0}(z)=-({C}_{max}-{C}_{min})\frac{z}{L+\pi R/2}+\frac{{C}_{max}+{C}_{min}}{2}$$

The above equation indicates that ${\lambda }_{D}$ is now a variant increasing from high salt concentration end through the channel to the lower one as seen in the inset of Fig. 1, since *C*_0_ decreases along the axis. On the other hand, in TMS model the ion concentrations are evaluated based on two conditions, one is a simplification of thermodynamic distribution and the other is the electroneutrality requirement:

4 $$\{\begin{array}{c}{C}_{+}{C}_{-}={C}_{0}^{2}\\ {C}_{+}-{C}_{-}=\frac{-2\pi R{\sigma }_{w}}{\pi {R}^{2}e}\end{array}$$

In the above, the radial nonuniformity of ionic concentration has been ignored $(\partial {C}_{\pm }/\partial r=0)$. In this way the TMS model significantly relieves the computation burden; however it is incompetent to evaluate several important quantities, since it neglects the variation of ion concentration along the channel radial direction. Hence in this work we employ the space-charge model (Detailed discussion is provided in Section Comparison with Teorell-Meyer-Sievers Model of the Supplementary Materials).

The electrical current along channel axis is then written as follows

5 $${I}_{z}=e(-{D}_{+}\frac{\partial {{\rm{\Lambda }}}_{+}}{\partial z}+{D}_{-}\frac{\partial {{\rm{\Lambda }}}_{-}}{\partial z})-e({\mu }_{+}{{\rm{\Lambda }}}_{+}+{\mu }_{-}{{\rm{\Lambda }}}_{-})\frac{\partial {V}_{0}}{\partial z}$$

where ${{\rm{\Lambda }}}_{\pm }$ is the line density of cations/anions along the channel axial direction:

6 $${{\rm{\Lambda }}}_{\pm }={C}_{0}(z\mathrm{)2}\pi {\int }_{0}^{R}\exp (\mp \bar{\varphi })rdr$$

#### ${\rm{\Delta }}{V}_{\sigma }$*versus*${\rm{\Delta }}{V}_{D}$: Skin and bulk effects

The relation between two experimentally measurable quantities, which are the longitudinal voltage *V*_0_ and the electrical current *I*_*z*_, is derived from Eq. 5:

7 $$\frac{\partial {V}_{0}}{\partial z}=\frac{{k}_{B}T}{e}\frac{{D}_{-}{{\rm{\Lambda }}}_{-}-{D}_{+}{{\rm{\Lambda }}}_{+}}{{D}_{-}{{\rm{\Lambda }}}_{-}+{D}_{+}{{\rm{\Lambda }}}_{+}}\frac{\partial \,\mathrm{ln}\,{C}_{0}}{\partial z}-{I}_{z}\frac{1}{{\mu }_{+}{{\rm{\Lambda }}}_{+}+{\mu }_{-}{{\rm{\Lambda }}}_{-}}$$

In the above derivation the approximation ${\partial }^{2}\bar{\phi }/\partial z\partial r\approx 0$ has been used. The open-circuit voltage ${\rm{\Delta }}{V}_{op}$ is then attained by noticing that ${I}_{z}=0$:

8 $$\frac{\partial {V}_{op}}{\partial z}=\frac{{k}_{B}T}{e}\frac{{D}_{-}{{\rm{\Lambda }}}_{-}-{D}_{+}{{\rm{\Lambda }}}_{+}}{{D}_{-}{{\rm{\Lambda }}}_{-}+{D}_{+}{{\rm{\Lambda }}}_{+}}\frac{\partial \,\mathrm{ln}\,{C}_{0}}{\partial z}$$

Traditionally, the coefficient $({D}_{+}{{\rm{\Lambda }}}_{+}-{D}_{-}{{\rm{\Lambda }}}_{-})/({D}_{-}{{\rm{\Lambda }}}_{-}+{D}_{+}{{\rm{\Lambda }}}_{+})$ is written with transference numbers of cations as $\mathrm{(2}{T}_{+}-\mathrm{1)}$, where ${T}_{\pm }={D}_{\pm }{{\rm{\Lambda }}}_{\pm }/({D}_{-}{{\rm{\Lambda }}}_{-}+{D}_{+}{{\rm{\Lambda }}}_{+})$ ^3,33^. The above equation further illustrates that both the difference between the cation and anion amounts and that between their diffusion coefficients would contribute to the emergence of open-circuit voltage, when a saline concentration gradient is imposed. In order to single out each effect, we define the Exclusion potential ${\rm{\Delta }}{V}_{\sigma }$ and Diffusion potential ${\rm{\Delta }}{V}_{D}$ as follows:

9 $$\frac{\partial {V}_{\sigma }}{\partial z}=\frac{{k}_{B}T}{e}\frac{{{\rm{\Lambda }}}_{-}-{{\rm{\Lambda }}}_{+}}{{{\rm{\Lambda }}}_{-}+{{\rm{\Lambda }}}_{+}}\frac{\partial \,\mathrm{ln}\,{C}_{0}}{\partial z}$$

10 $$\frac{\partial {V}_{D}}{\partial z}=\frac{{k}_{B}T}{e}\frac{{D}_{-}-{D}_{+}}{{D}_{-}+{D}_{+}}\frac{\partial \,\mathrm{ln}\,{C}_{0}}{\partial z}$$

Through rewriting in the above formats, the physical origin of the saline concentration induced voltage in nanochannels is demonstrated explicitly: ${V}_{\sigma }$ is caused by the extra-ions induced by the channel wall surface charges, since $({{\rm{\Lambda }}}_{-}-{{\rm{\Lambda }}}_{+})$ is determined by ${\sigma }_{w}$; ${V}_{D}$ is by the difference between cation and anion mobility in the aqueous environment. From the above equations, we also find that the two components of ${V}_{op}$, ${V}_{\sigma }$ and *V*_*D*_, are formally quite similar. Both coefficients, $({{\rm{\Lambda }}}_{-}-{{\rm{\Lambda }}}_{+})/({{\rm{\Lambda }}}_{-}+{{\rm{\Lambda }}}_{+})$ and $({D}_{-}-{D}_{+})/({D}_{-}+{D}_{+})$, are in the range from −1 to 1. The fact points out that the magnitudes of ${\rm{\Delta }}{V}_{\sigma }$ and ${\rm{\Delta }}{V}_{D}$ can be comparable when the ${\sigma }_{w}$ -induced and $({D}_{-}-{D}_{+})$ -induced effects fall in the same orders of magnitude. As we are going to see, it is the mechanism that accounts for the observed variation of ${\rm{\Delta }}{V}_{op}$ with changing nanochannel parameters such as the amplitudes and species of salt concentration bias.

**Figure 2:**
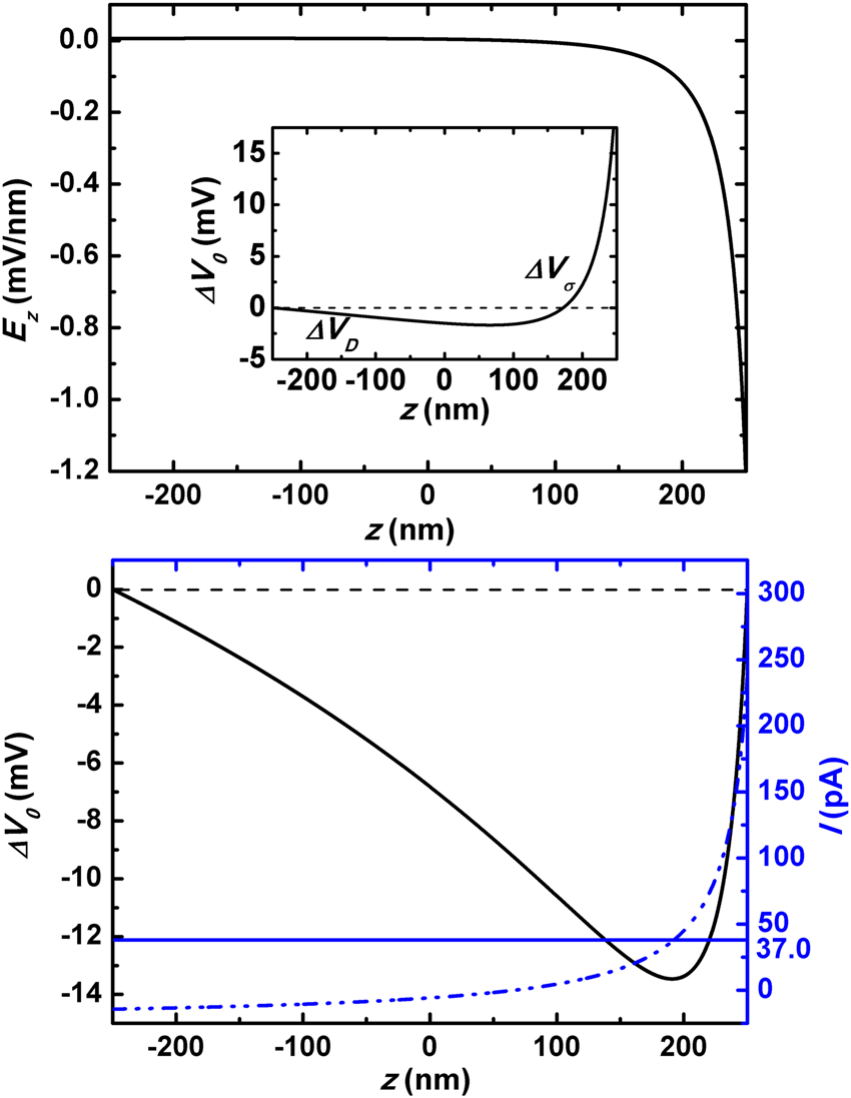
(**a**) Open-circuit state: the channel-axial distribution of *z*-component electrical field ${E}_{z}(z)$ in a *R* = 10 nm and *L* = 500 nm nanochannel, where a NaCl concentration bias ${C}_{max}/{C}_{min}$ = 600 mM/10 mM is imposed at the two ends and the surface charge density on the wall ${\sigma }_{w}=-50$ mC/m^3^. Inset plots the corresponding self-built voltage *V*(*z*). The symbols ${\rm{\Delta }}{V}_{D}$ and ${\rm{\Delta }}{V}_{\sigma }$ indicate regions dominating by the diffusive potential and by exclusion potential, respectively. (**b**) Short-circuit state: the left coordinate demonstrates the calculated distribution of electromotive voltage along the channel axis *V*(*z*) for the same nanochannel as Fig. 2a; the right one shows the diffusive component of the electrical current shown in Eq. 5 (dash-dot line), and the overall electrical current (real line).

In the real experiments, both the open-circuit voltage ${\rm{\Delta }}{V}_{op}$ and short-circuit current *I*_*sh*_ are important indexes measuring the electrical properties of saline concentration biased nanochannels. Figure 2a and the inset plot the distributions of open-circuit state electrical field *E*_*z*_ and electromotive potential *V*_0_ along the channel axis inside a $R=10$ nm and $L=500$ nm nanochannel with density ${\sigma }_{w}=-50$ mC/m^2^ (the parameters come from the related experiments)^3,18^. It clearly demonstrates that around the saline end ($z=-L/2$) the diffusion effect dominates while at the opposite end the exclusion effect takes control (More discussions about two extreme cases are provided in Section Comparison with Two Extreme Cases of the Supplementary Material for further demonstration). By resorting to the above ${\rm{\Delta }}{V}_{\sigma }$ *versus* ${\rm{\Delta }}{V}_{D}$ picture, we show that several interesting and yet puzzling experimental observations are now interpreted satisfactorily. One discovery is the first increasing and then decreasing magnitude of ${\rm{\Delta }}{V}_{op}$ with increasing electrolyte concentration at *either* end of the channel^3^. In our previous work, we discussed the varying trend of ${\rm{\Delta }}{V}_{op}$ with changing saline concentration at the saline end ${C}_{max}$ ^34^. We showed that for small saline concentrations, the major effect of increasing ${C}_{max}$ is the enhanced diffusion flux of the extra cations and thus an increased exclusion voltage ${\rm{\Delta }}{V}_{\sigma }$ is attained; however, after a turning point of ${C}_{max}$ value, the substantially decreased EDL thickness with larger ${C}_{max}$ causes the exclusion effect to be more skinny, and thereby leads to less ${\rm{\Delta }}{V}_{\sigma }$. In this way the experimentally observed ${\rm{\Lambda }}$-shape change of ${\rm{\Delta }}{V}_{op}$ was well understood in our theoretical framework of competition between ${\rm{\Delta }}{V}_{\sigma }$ and ${\rm{\Delta }}{V}_{D}$. From the viewpoint of application, the above analysis elucidates that a naive design by imposing directly the large NaCl concentration difference between sea and river water to the nanochannel system can not yield the optimal output power or efficiency. Instead, an improved device architecture is called for as we are going to demonstrate later.

In Fig. 2b we demonstrate the calculated short-circuit state quantities in a *R* = 10 nm and *L* = 500 nm nanochannel biased by sea and water solutions (NaCl with ${C}_{max}/{C}_{min}=$ 600 mM/10 mM). The blue dash-dot line plots the diffusive component of electrical current, which is the first term shown in Eq. 5. It changes sign from the saline end to the dilute one. At ${C}_{max}$ end of the channel ($z=-L\mathrm{/2}$), the diffusion contribution to the electrical current points from ${C}_{min}$ to ${C}_{max}$. This is attributed to two facts: on one side, the diffusive coefficient of Cl^−^ is about one time larger than that of Na^+^ (${D}_{-} > {D}_{+}$); on the other side, the concentrations of the two species are nearly the same (${{\rm{\Lambda }}}_{-}\approx {{\rm{\Lambda }}}_{+}$) since the amount of ${\sigma }_{w}$-induced excessive counterions is much smaller than the imposed bulk concentration there ($|{{\rm{\Lambda }}}_{+}-{{\rm{\Lambda }}}_{-}|\ll {{\rm{\Lambda }}}_{\pm }\approx {C}_{max}$). The consequence is that the magnitude of anionic diffusion flow outweighs that of cationic one at ${C}_{max}$ end, and the overall diffusive component of electrical current heads towards ${C}_{max}$ end. However, at ${C}_{min}$ end of the channel ($z=L\mathrm{/2}$), the direction of the diffusive contribution becomes reversed. This time the quantity of counterions dominates (${{\rm{\Lambda }}}_{+}-{{\rm{\Lambda }}}_{-}\gg |{C}_{0}\pi {R}^{2}|$) due to the quite dilute saline concentration there. Therefore the diffusive current turns to be cationic.

From the above discussion, we are aware that the diffusive electrical current varies significantly along the channel axis. In order to keep the continuity of the overall electrical current, the electrical field has to take a self-adaption so that the variation of electrophoretic component of ion current compensates the changes of diffusive one. This interprets the shape of ${\rm{\Delta }}V$ shown by black line in Fig. 2b: at the left part of the channel its derivative ${E}_{z}$ points from ${C}_{max}$ end to ${C}_{min}$ one, while at the right part the electrical field heads towards the opposite direction. Such an electrical field results in conversely oriented electrophoretic current at the two sides of the channel, and in this manner the sum of diffusive and electromigration parts of current keeps invariant along the channel axis.

## Results and Discussion

### Comparison with Experiments

In this section, we are going to discuss the nanochannel RED observed in several experiments and demonstrate that the established model in the above section can interpret the experimental reports satisfactorily.

#### Dependence on Ion Species

**Figure 3:**
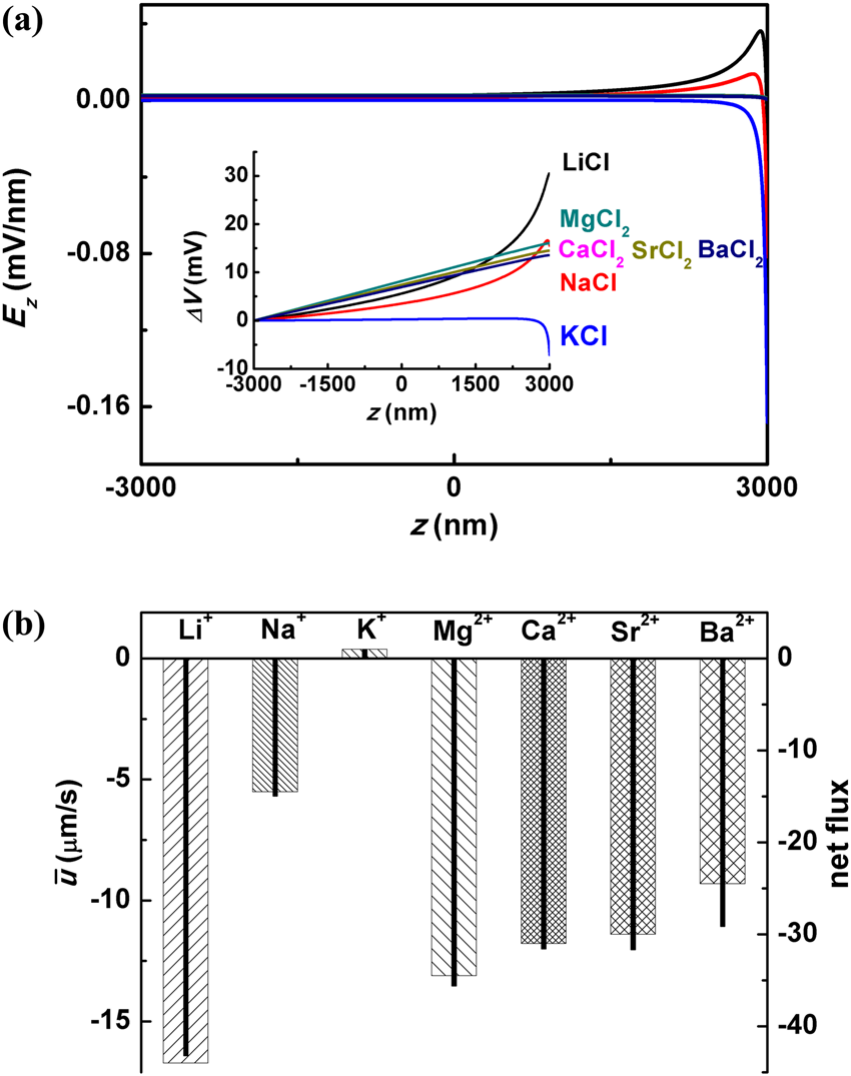
(**a**) The channel axial distribution of self-built electrical field ${E}_{z}(z)$ in a $R=50$ nm and $L=6$ $\mu$m nanochannel under open-circuit state when various types of salt are imposed. The corresponding voltage distribution ${\rm{\Delta }}V(z)$ is plotted in the inset. Here the parameters are set according to the experiments^35^. The surface charge density is set as ${\sigma }_{w}=-0.8$ mC/m^3^ ^34^. (**b**) The columns and real lines represent the experimentally measured net flux (right)^35^ and theoretically calculated average flow speed ${\bar{u}}_{z}$ by our model.

Yang *et al*. reported that both the direction and magnitude of saline gradient induced fluid showed strong dependence on the types of imposed electrolyte^35^. As demonstrated by our previous work, the experimentally observed phenomenon was interpreted as electroosmotic flow (EOF) stimulated by open-circuit RED in nanochannels^34^. Moreover, Fig. 3 illustrates that even given the same concentrations of imposed chlorides at the saline end, the orientations and amplitudes of the induced open-state voltages ${\rm{\Delta }}{V}_{op}$ would be quite different depending on the cation types as shown in Fig. 5 of ref.^35^. We are going to show that it can be well understood in our theoretical framework of competition between ${\rm{\Delta }}{V}_{\sigma }$ and ${\rm{\Delta }}{V}_{D}$.

For monovalent chlorides, the experiments showed that ${({\rm{\Delta }}V)}_{{\rm{LiCl}}} > {({\rm{\Delta }}V)}_{{\rm{NaCl}}} > 0 > {({\rm{\Delta }}V)}_{{\rm{KCl}}}$; while for divalent chloride the relation was ${({\rm{\Delta }}V)}_{{{\rm{MgCl}}}_{{\rm{2}}}} > {({\rm{\Delta }}V)}_{{{\rm{CaCl}}}_{{\rm{2}}}}\approx {({\rm{\Delta }}V)}_{{{\rm{SrCl}}}_{{\rm{2}}}} > {({\rm{\Delta }}V)}_{{{\rm{BaCl}}}_{{\rm{2}}}}$. As illustrated by the inset of Fig. 1, the direction of electrical field by exclusion effect is opposite to that by diffusion effect, when negatively charged wall (${\sigma }_{w} < 0$) and chlorides (Cl^−^) are employed. Here the physical mechanism is that the diffusion coefficients of various kinds of cations are usually smaller than that of Cl^-^. In other words, Cl^−^ ions diffuse faster than the cations from the concentrated end to the diluter one. Therefore, the electrical field ${\vec{E}}_{D}$ established through this mobility difference points from ${C}_{max}$ end to ${C}_{min}$ one. On the other hand, ${\vec{E}}_{\sigma }$ by exclusion effect orients in the contrary direction due to the cation-selective properties of the channel wall. Which factor dominates is determined by the competition between ${\rm{\Delta }}{V}_{D}$ and ${\rm{\Delta }}{V}_{\sigma }$. For monovalent salt the diffusion potential ${\rm{\Delta }}{V}_{D}$ by LiCl ranks the largest, that by NaCl the second and KCl the smallest, since ${D}_{{\rm{Li}}}/{D}_{{\rm{Cl}}}$ = 0.51, ${D}_{{\rm{Na}}}/{D}_{{\rm{Cl}}}$ = 0.66 and ${D}_{{\rm{K}}}/{D}_{{\rm{Cl}}}$ = 0.96. On the other side, the exclusion effect was significantly suppressed in the experiments when the imposed chloride concentration at the saline end was ${C}_{{\rm{\max }}}$ = 50 mM. The physical mechanism is that the EDL thickness was almost negligible compared to the channel radius $R$ = 50 nm (${\lambda }_{D}\,\approx$ 1.3 nm) at the saline end of the channel. Only for KCl where the diffusion effect was also quite weak, the overall self-built ${\rm{\Delta }}V$ exhibited exclusion property. It interprets why ${({\rm{\Delta }}V)}_{{\rm{LiCl}}} > {({\rm{\Delta }}V)}_{{\rm{NaCl}}} > 0$ while ${({\rm{\Delta }}V)}_{{\rm{KCl}}} < 0$. Then, similar analysis is applicable to the situations of divalent chloride concentration bias where ${D}_{{\rm{Mg}}}/{D}_{{\rm{Cl}}}$ = 0.35, ${D}_{{\rm{Ca}}}/{D}_{{\rm{Cl}}}\approx {D}_{{\rm{Sr}}}/{D}_{{\rm{Cl}}}$ = 0.39 and ${D}_{{\rm{Ba}}}/{D}_{{\rm{Cl}}}$ = 0.42.

The above mechanisms are further manifested by the quantitative calculation and demonstration of electromotive potential along channel axis *V*_0_(*z*) under various kinds of salt, as shown in Fig. 3a. Accordingly, the experimentally measured values of ${C}_{{\rm{\max }}}/{C}_{{\rm{\min }}}$ -induced net flow are plotted with columns in Fig. 3b, while the simulation results are by real lines as comparison. We conclude that our *exclusion versus diffusion* model interprets the saline species-dependent phenomena in the nanochannel RED experiments. Moreover, from the viewpoint of energy converting, we find that by using SiO_2_ the energy converting efficiency is reduced due to the conflict between diffusion effect and exclusion one. In contrary, The cationic surface charges on Al_2_O_3_ channel wall give rise to accordantly oriented ${\rm{\Delta }}{V}_{\sigma }$ and ${\rm{\Delta }}{V}_{D}$, and therefore the overall open-circuit voltage is enhanced^19^. However, SiO_2_ also has advantage in the matured process and being easy to achieve large-scale integrated circuits.

#### Dependence on ${C}_{max}/{C}_{min}$

**Figure 4:**
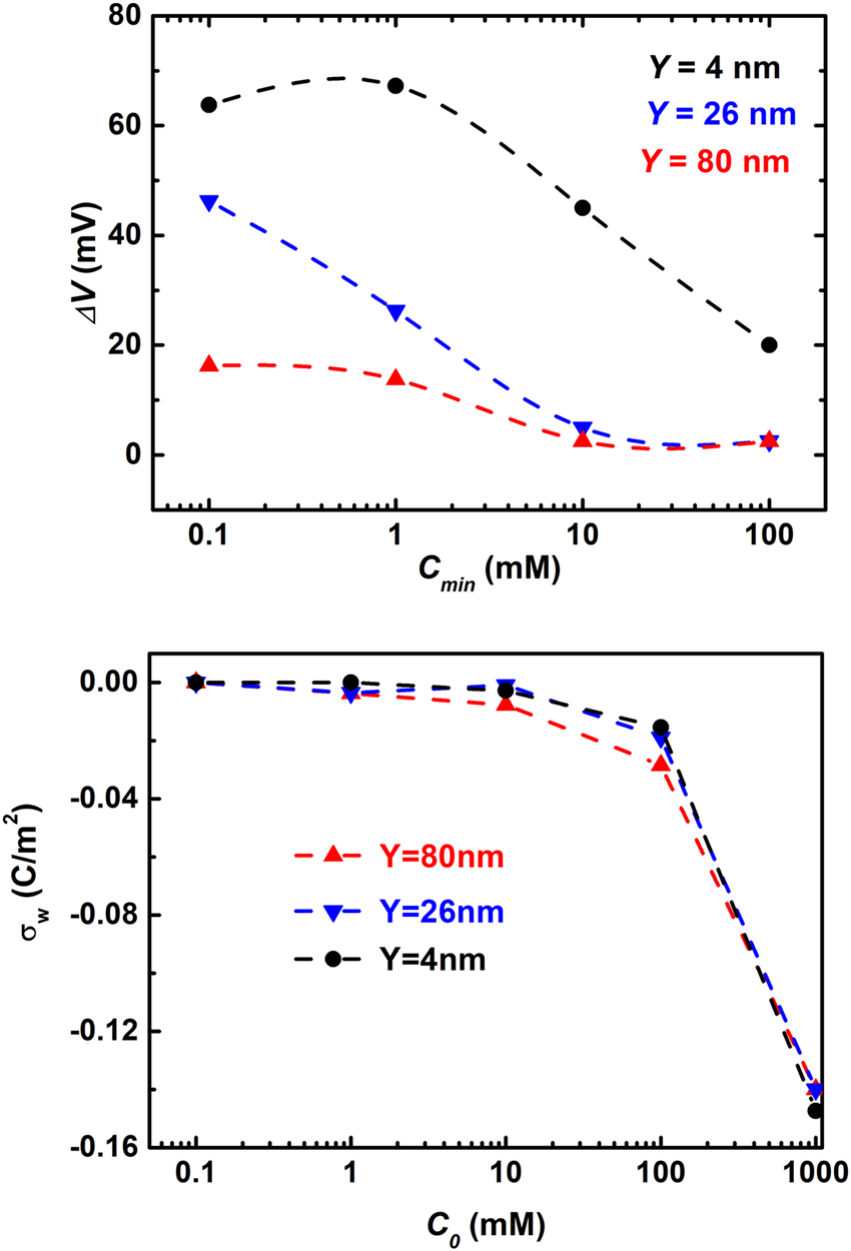
(**a**) The experimentally measured open-circuit voltages (as shown in symbols) and the calculated open-circuit voltages (as shown in dashed lines) under increasing concentration of KCl input at the dilute end ${\rm{\Delta }}{V}_{op}({C}_{min})$ ^3^. (**b**) The calculated density of surface charges on the channel wall ${\sigma }_{w}$ as a function of the imposed KCl concentration *C*_0_, by fitting fitting the above experiments.

The first increasing and then decreasing magnitude of ${\rm{\Delta }}{V}_{op}$ with increasing electrolyte concentration at either end of the channel, was observed by experimental study^3^. It indicates again that a naive design by imposing directly the large salt concentration difference between sea and river water to the nanochannel system may not yield the optimal output power or efficiency. In other words, an improved device architecture for enhancing energy harvesting performance is called for, and first of all, an analysis of the related physical mechanism is essential. In our previous work, we have illustrated the physical mechanisms for the ${\rm{\Lambda }}$-shape turning of the exclusion potential ${\rm{\Delta }}{V}_{\sigma }$ with the increasing salt concentration ${C}_{max}$ ^34^, as observed in other experiments^35^. The large open-circuit voltage is induced under very low concentration ${C}_{min}$ even in relatively large nanopores(the Y = 80 nm in the Fig. 4a). It’s can be explained by our physical picture: On the one hand, the EDL is much thicker in 0.1 mM KCl solutions, where the Debye length ${\lambda }_{D}=\sqrt{{\varepsilon }_{f}{K}_{B}T/2{C}_{0}{e}^{2}}\approx 30.4\,nm$. Thus the EDL overlap even in relatively large nanopores. Such strong ion selectivity will lead to large amplitude of the exclusion voltage. On the other hand, the bigger salt concentration difference ${\rm{\Delta }}C=({C}_{max}-{C}_{min})$ in 0.1 mM KCl solutions, the stronger diffusion flux of the ions, which results in larger diffusion voltage. The sum up of reinforced exclusion voltage and diffusion voltage give rise to the large open-circuit voltage even in relatively large nanopores. However, the experiments discussed here further demonstrated that the initial increasing of salt concentration at the diluter end, ${C}_{min}$, would also boost a short increasing trend of the open-circuit potential $\Delta {V}_{op}$ (Black symbols shown in Fig. 4a). At first glance, it seemed to conflict with the rationale that fixed ${C}_{max}$ and increasing ${C}_{min}$ would result in two consequences, and both of them lead to attenuated ${\rm{\Delta }}{V}_{op}$. One is that the smaller salt concentration difference ${\rm{\Delta }}C=({C}_{max}-{C}_{min})$, the weaker diffusion flux of the extra cations. The other is the thinner EDL with increasing ${C}_{min}$, which leads to weaker ion selectivity of the channel and thus smaller exclusion potential. Here we attribute the first-stage increasing behavior of ${\rm{\Delta }}V({C}_{min})$ to the dependence of wall surface charge density ${\sigma }_{w}$ on the local salt concentration *C*_0_. The charges on the SiO_2_ surface used in the experiments^3^ are thought to come from chemical reaction

11 $${\rm{SiOH}}\rightleftharpoons {{\rm{SiO}}}^{-}+{{\rm{H}}}^{+}$$

Let us first suppose invariant concentrations of SiO^−^ under various bulk concentrations of saline. The different capability of shielding SiO^-^ under different saline concentrations would result in variant magnitudes of electrostatic potentials ${\varphi }_{s}$ at the channel/liquid interface. The consequence is that concentrated electrolyte leads to smaller surface voltage (see for example the calculation and Fig. 2 in our previous work)^36^:

12 $${C}_{0}\uparrow \Rightarrow {\varphi }_{s}\downarrow $$

It further leads to smaller proton concentration near the channel surface ${[{H}^{+}]}_{s}$, according to the thermodynamic distribution along the channel radial direction (notice that ${\varphi }_{s}$ is negative due to the anionic properties of channel surface).

13 $${[{H}^{+}]}_{s}={[{H}^{+}]}_{b}\exp (\frac{-e{\varphi }_{s}}{kT})$$

In the above, ${[{H}^{+}]}_{b}$ is the bulk concentration of protons determined by system pH. In line with the above changes, the chemical reaction has to move towards the production of more SiO^−^ when larger concentration of electrolyte is imposed:

14 $$\frac{{[{H}^{+}]}_{s}{{\rm{\Gamma }}}_{{{\rm{SiO}}}^{-}}}{{{\rm{\Gamma }}}_{{\rm{SiOH}}}}={10}^{-pK}{\rm{M}}$$

The above analysis indicates that increasing ${C}_{min}$ on one hand attenuates the saline concentration bias ${\rm{\Delta }}C$ across the channel, while on the other hand it reinforces the ion selectivity of channel through increased surface charge density ${\sigma }_{w}$.

15 $${C}_{0}\uparrow \Rightarrow {\sigma }_{w}\uparrow $$

The overall effect on the variation trend of ${\rm{\Delta }}{V}_{\sigma }({C}_{min})$ is determined by the relative strengths between the two factors.

Figure 4a plots the experimentally reported self-built voltage ${\rm{\Delta }}{V}_{0}$ as a function of the imposed KCl concentration at the diluter end ${C}_{min}$, in nanochannels with various heights. Figure 4b plots the corresponding surface charge density ${\sigma }_{w}$ fitted by our model. The varying trends of ${\sigma }_{w}({C}_{0})$ show nice agreement with our analysis based on the charge regulation model. However, the quantitatively calculated ${\sigma }_{w}({C}_{0})$ by the charge regulation model are 1 or 2 orders smaller, as shown in Fig. S5 of the Supplementary Materials. The quantities are also 1 or 2 orders smaller than that estimated via other experiments^18^. We have given some discussion in the new section **Comparison with Charge Regulation** Model in the Supplemental Materials, and left further exploring to interested readers.

#### Conical Nanochannels

**Figure 5:**
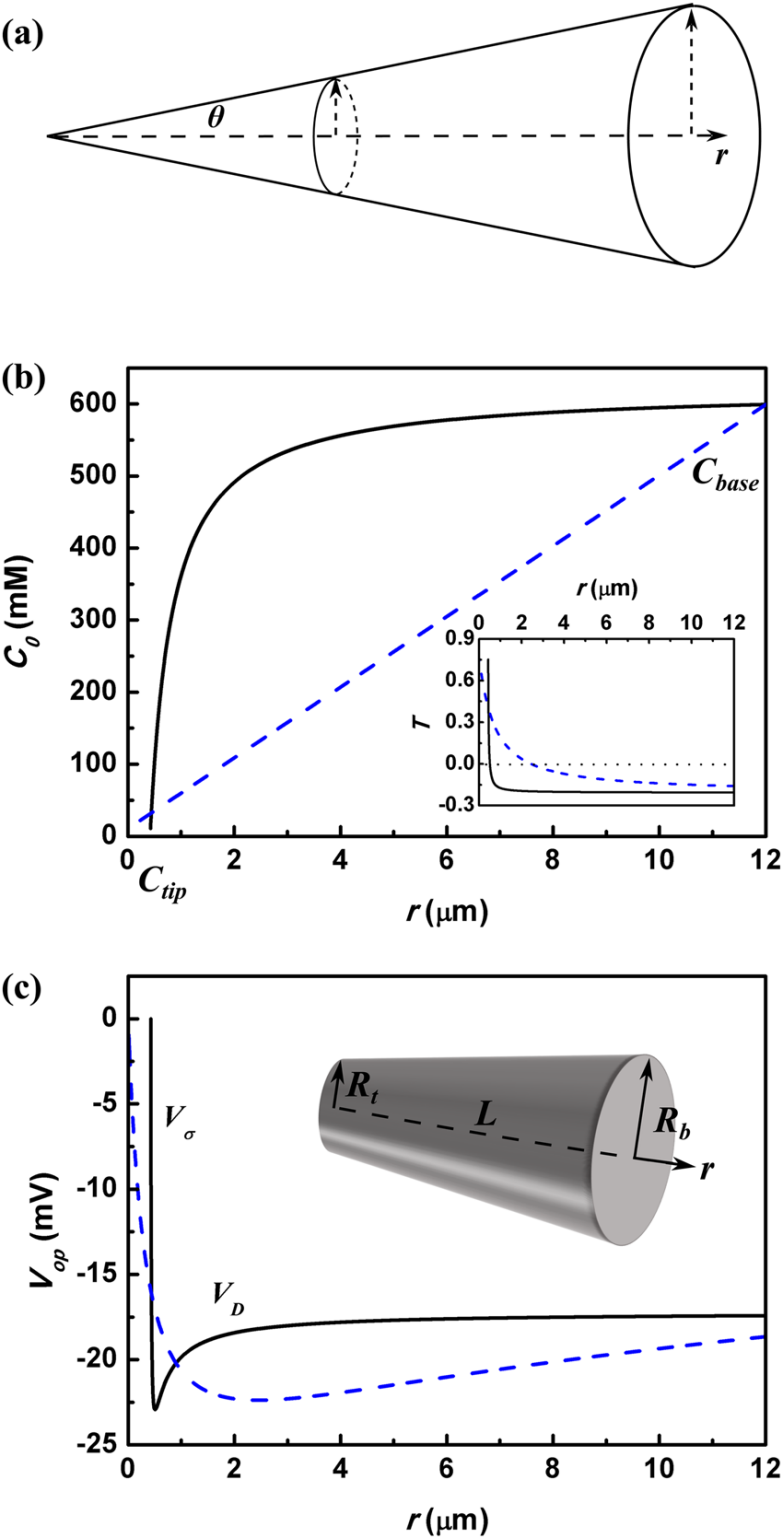
(**a**) Schematic diagram of conical nanochannel. (**b**) The NaCl concentration distribution *C*_0_ along the channel axis for conical-shaped nanochannel (black line). Inset plots the transmission number along the axis. Here the parameters are from the experiments^18^: ${R}_{t}=20.5$ nm, ${R}_{b}=600$ nm, $L=12\mu$m and ${\sigma }_{w}=-60$ mC/m^2^. The concentration drop within in a cylinder (blue line) nanochannel with $R=20.5$ nm and $L=12\mu$m is plotted with blue line as a comparison. (**c**) The voltage distribution along the channel axis. Inset is the schematic view of conical-shaped nanochannel, where the length *L*, tip and base radii, *R*_*t*_ and *R*_*b*_ are characterized.

Recent experiments demonstrated that by using conical nanochannels as shown schematically in Fig. 5a, the maximum output electrical power with a *single* channel could approach tens of picowatts in the presence of the KCl concentration bias^18^. By imposing KCl with small concentration at the tip end while large concentration at the base one (${C}_{t}/{C}_{b}=1$ mM/10 mM), the open-circuit voltage ${\rm{\Delta }}{V}_{op}$ and short-circuit current *I*_*sh*_ measured in the system reached as large as 206 mV and 52.7 pA. The results might illuminate a prospect of powering nanoelectronic, optoelectronic or tiny biomedical devices with nanochannel RED power source. Hence understanding the dependence of output electrical power on the nanochannel shape is essential, so that potential improvements of the performance may be achieved by designing the channel geometry. Theoretical studies based on a 2-dimensional multiphysical model also confirmed that ${\rm{\Delta }}{V}_{op}$ and *I*_*sh*_ would be enhanced in the conical nanochannel systems^24^. Here we give a brief analysis why the conical shape facilitates the current and voltage in the nanochannel RED, and whether the conical shape really improves the indexes of performance.

The conical nanochannel system is described within the spherical coordinate^37^

16 $${\theta }_{m}={\rm{arc}}\,\tan (\frac{{r}_{b}-{r}_{t}}{L})$$

In the above *R*_*t*_, *R*_*b*_ and *L* are the radii of the tip and base ends, and the length of the channel as seen in Fig. 5a and the inset of Fig. 5c (Be ware that now *r* is the channel axial direction). The saline concentration distribution along the channel axis is derived from the requirement of ion flow continuum $\nabla \cdot {\vec{J}}_{i}=0$:

17 $$\frac{\partial }{\partial r}[{D}_{i}\frac{\partial {C}_{0}}{\partial r}2\pi {r}^{2}\mathrm{(1}=-\cos \,{\theta }_{m})]=0$$

where ${r}_{t}cot\,{\theta }_{m}\le r\le {r}_{b}\,\cot \,{\theta }_{m}$. The boundary conditions at the two ends of the conical channel reads as follows:

18 $$\{\begin{array}{c}{C}_{0}(r{)|}_{r={r}_{b}\cot {\theta }_{m}}={C}_{b}\\ {C}_{0}(r{)|}_{r={r}_{t}\cot {\theta }_{m}}={C}_{t}\end{array}$$

In the above, *C*_*b*_ and *C*_*t*_ are the concentrations of salt imposed at the base and tip ends, respectively. We then arrive at

19 $${C}_{0}(r)=({C}_{t}-{C}_{b})\frac{{r}_{t}\,{r}_{b}}{({r}_{b}-{r}_{t})r}\,\cot \,{\theta }_{m}+\frac{{C}_{b}{r}_{b}-{C}_{t}{r}_{t}}{{r}_{b}-{r}_{t}}$$

The Poison-Boltzmann equation for calculating the electrostatic potential $\varphi$ is written in the spherical coordinate as follows:

20 $$\{\begin{array}{c}\frac{1}{{r}^{2}\,\sin \,\theta }\frac{\partial }{\partial \theta }(\sin \,\theta \frac{\partial \bar{\varphi }(r,\theta )}{\partial \theta })=\frac{\sin \,{\rm{h}}(\bar{\varphi }(r,\theta ))}{{\lambda }_{D}^{2}}\\ {\begin{array}{c}\frac{\partial \bar{\varphi }(r,\theta )}{\partial \theta }\end{array}|}_{\theta =0}=0\\ {\begin{array}{c}\frac{\partial \bar{\varphi }(r,\theta )}{\partial \theta }\end{array}|}_{\theta ={\theta }_{m}}=\frac{e{\sigma }_{w}}{\varepsilon {k}_{B}T}r\end{array}$$

The line density of ions along the channel axial direction is then evaluated by

21 $${{\rm{\Lambda }}}_{\pm }(r)=2\pi {r}^{2}{C}_{0}(r){\int }_{0}^{{\theta }_{m}}\exp [\mp \bar{\varphi }(r,\theta )]\sin \,\theta \,d\theta $$

In the calculation, we numerically solve the above equation and obtain the concentration distribution of cations and anions in the system. The open-circuit voltage and short-circuit electrical current are then calculated with similar approach as shown for cylinder nanochannels.

We assume that a NaCl concentration bias ${C}_{b}/{C}_{t}=600$ mM/10 mM between the sea and water is put into the base and tip ends of the experimentally fabricated conical nanochannel system, where ${R}_{t}=20.5$ nm, ${R}_{b}=600$ nm, $L=12$ $\mu$m and ${\sigma }_{w}=-60$ mC/m^2^ ^18^. The calculated ion concentration distribution along the channel axial direction is plotted in Fig. 5b. As a comparison, we further plot the ion concentration landscape in a cylinder nanochannel where $R={R}_{t}$, $L=12\,\mu$  m and ${\sigma }_{w}=-60$ mC/m^2^. The figure illustrates that a steeper concentration drop would be resulted in around the tip end of the conical nanochannel. In other words, the amplitude of diffusion flux is larger at the tip end than that at the base one. This is ascribed to the requirement of ion current conservation (${I}_{\pm }\approx A{D}_{\pm }\partial {C}_{0}/\partial r$), since at the tip the cross-section *A* is much smaller. The physical consequence is that the EDL becomes thinner in the conical nanochannels than in the cylinder one. The attenuated ion selectivity thus interprets the slightly smaller open-circuit voltage ${\rm{\Delta }}{V}_{op}$ in the conical nanochannels, as shown in Fig. 5c. On the other hand, the gradually increasing radius from the tip end to the base one of the conical nanochannel leads to a much smaller resistance compared to that of a cylinder nanochannel, given the same tip-end radii. This is the physical origin why a much larger short-circuit electrical current *I*_*sh*_ is achieved in the conically shaped nanochannels, as seen in Table 1. The table further demonstrates that as individual channels, the conical ones will harvest output electrical power several tens of times larger than the cylinder counterparts. However, the power density of the conical ones *P*/*A*, which is the more crucial index for mass production, turns to be orders smaller than that of the cylinder ones (Here $A=\pi {R}_{b}^{2}$ for conical nanochannels). The physical mechanisms are clearly demonstrated by comparing the values of input thermal power ${P}_{th}$ for the two differently shaped nanochannels in the table. As we analyzed previously, the gradually increasing radius from the tip end to the base one of the conical channel would lead to larger diffusion flux of ions. However, the thermal power ${P}_{th}$ per-area gained by the conical-shaped nanochannel is reduced. Therefore, our conclusion is that by fabricating conical shape nanochannels, the output power of *individual* channels can be enhanced comparing to the cylinder ones; however, the power density is significantly reduced and thus it is not suitable for mass fabrication and integration.

**Table 1:** Performance comparison between conical and cylinder nanochannels.

Type	*V*_*op*_ (mV)	*I*_*sh*_ (pA)	*P* (pW)	*P*/*A* (W/m ^2^)	*P*_*th*_ (pW)	*γ*(%)
Conical	17.4	128	0.558	0.494	839	0.0666
Cylinder	18.8	4.83	0.0227	17.2	199	0.0114

Before ending this section, we stress that the above electrokinetic model is established based on the approximation of decoupling the channel axial and radial transport. Compared to a straightforward full-dimensional modeling and then numerical calculation^18,25^, the major advantage of our approach is that the physical mechanisms can be clearly elucidated. Not only has the existence of exclusion and diffusion potentials ${\rm{\Delta }}{V}_{\sigma }$ and ${\rm{\Delta }}{V}_{D}$ been outlined from the the expressions of self-built voltage, but also the competing roles of the two mechanisms in determining the ion transport and their dependence on the device parameters have been demonstrated explicitly. By contrast, the full-dimensional numerical simulation may be more accurate for short-length nanochannels^38,39^, however at the expense of difficulty to illustrate the internal pictures since the longitudinal and transverse transport are coupled together. On the other side, the widely used TMS model treats the channel axial and radial electrostatics and ion movement separately in a similar way as space-charge model, and thus illustrative expressions can also be obtained for demonstrating mechanisms. However, the variation of the ion distribution along channel radial direction is absolutely neglected in TMS model $(\partial {C}_{\pm }/\partial r=0)$ while it has been considered in our space-charge one as see in Eq. 2. The consequence is that TMS model becomes incompetent when evaluating electroosmotic flow, current-voltage characteristic or the energy converting efficiency, as we have seen in the above(More details about the results of space-charge model and Teorell-Meyer-Sievers Model are provided in Fig. S3 of the Supplementary Materials). In conclusion, the space charge model employed in this work can achieve a nice balance between the requirements of physics illustration and quantitative accuracy.

### Energy conversion by RED in nanochannels

#### Two Indexes of Performance: Converting Efficiency and Power Density

Experimentally, linear current-voltage characteristic has been observed in saline gradient biased nanochannels made by various kinds of material^3,19^. The behavior can be deduced from our modelling shown in Eq. 7 by noticing $\partial {I}_{z}/\partial z=0$:

22 $${V}_{out}={\rm{\Delta }}{V}_{op}-{I}_{z}{R}_{ch}$$

where

23 $${R}_{ch}={\int }_{z=-L/2}^{z=L/2}\frac{1}{e({\mu }_{+}{{\rm{\Lambda }}}_{+}+{\mu }_{-}{{\rm{\Lambda }}}_{-})}dz$$

The above formula indicates that the equivalent circuit of nanochannel RED is a voltage source and a resistor in series. It interprets the linear *I*(*V*) curves measured in the experiments. From the viewpoint of application, the maximum output power density and the energy converting efficiency are the two crucial indexes characterizing the performance of nanochannel RED based energy harvesting. Below we show theoretical analysis and explore methods to enhance the indexes. First, the maximum output power is achieved when the load resistance *R*_*L*_ is the same as *R*_*ch*_

24 $${P}_{E}{|}_{max}=\frac{{\rm{\Delta }}{V}_{op}^{2}}{4\,{R}_{ch}}$$

where the output voltage is half the open-circuit one ${V}_{out}={\rm{\Delta }}{V}_{op}/2$. On the other side, the Gibbs free energy of mixing solutions with different salt concentrations is estimated as follows^7^:

25 $$-\frac{{\rm{\Delta }}{G}_{mix}}{\upsilon RT}={C}_{M}\,\mathrm{ln}({C}_{M})-\varphi {C}_{min}\,\mathrm{ln}({C}_{min})-\mathrm{(1}-\varphi ){C}_{max}\,\mathrm{ln}({C}_{max})$$

In the above, ${\rm{\Delta }}{G}_{mix}$ is the change of Gibbs free energy upon the mixing, $\upsilon$ is the number of ions the electrolyte molecule dissociates into, *R* is gas constant, *T* is the temperature, ${C}_{M}$, ${C}_{min}$ and ${C}_{max}$ are the molar salt concentrations of the aqueous solutions of mixture, diluter reservoir and saline one, and $\varphi$ is the ratio of total moles of solution from saline sea to dilute river in the system. For the monovalent ion system, the thermal power generated by mixing two solutions with different saline concentrations is then written as follows

26 $${P}_{th}=RT[{C}_{max}\,\mathrm{ln}({C}_{max})-{C}_{min}\,\mathrm{ln}({C}_{min})]\frac{{Q}_{+}+{Q}_{-}}{{C}_{max}}$$

where ${Q}_{\pm }$ is the cationic/annionic flow through the channel and according to our space charge model it is evaluated as

27 $${Q}_{\pm }=-{D}_{\pm }{{\rm{\Lambda }}}_{\pm }\frac{\partial \,\mathrm{ln}\,{C}_{0}}{\partial z}\mp {\mu }_{\pm }{{\rm{\Lambda }}}_{\pm }\frac{\partial V}{\partial z}$$

The energy converting efficiency is then defined as

28 $$\gamma =\frac{{P}_{E}}{{P}_{th}}$$

Here we call special attention to that the total flux of cations and anions *Q* is not proportional to the electrical current *I* by ions, since $Q=({Q}_{+}+{Q}_{-})$ while $I=e({Q}_{+}-{Q}_{-})$. In other words, *Q* cannot be derived simply from the expression for electrical current $I=({\rm{\Delta }}{V}_{op}-{V}_{out})/{R}_{ch}$. Instead, the channel axial distribution of the electrical field $(-\partial V/\partial z)$ has to be identified based on the conservation requirement $\partial {Q}_{\pm }/\partial z=0$. In our evaluation, we solve the above equations in a self-consistent way and then obtain the quantities such as $I({V}_{out})$.

**Figure 6:**
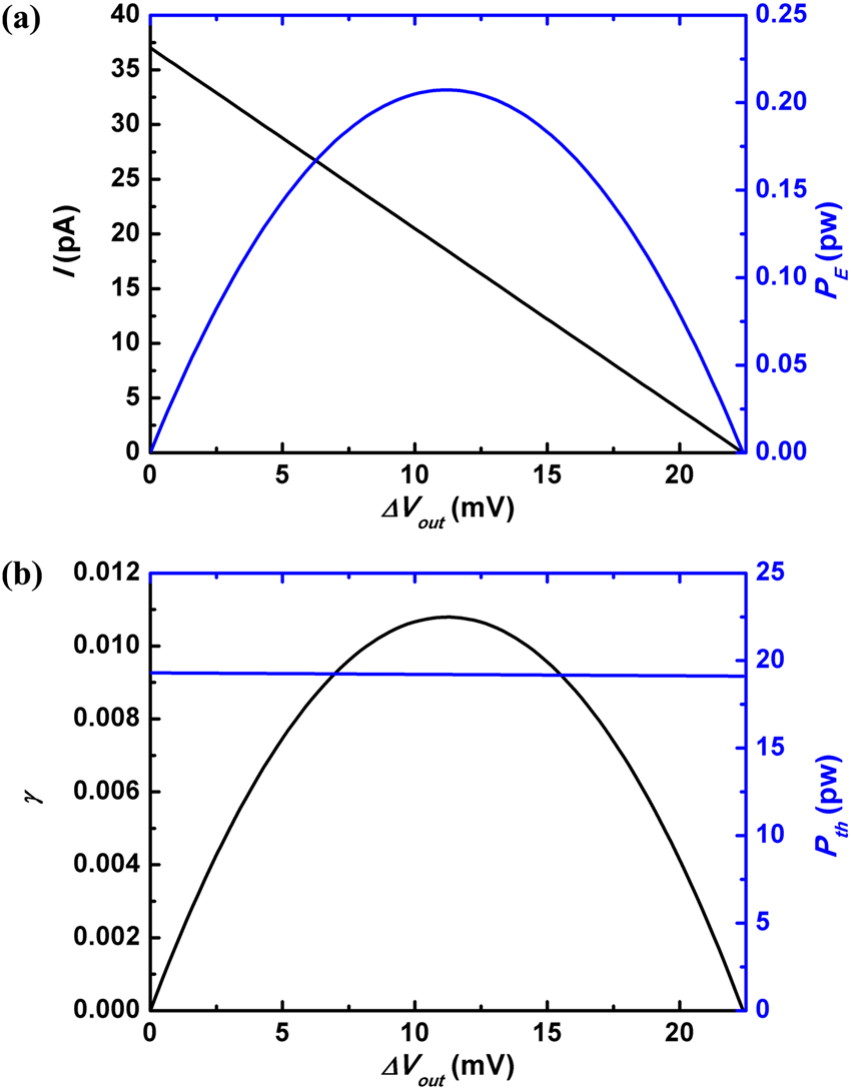
(**a**) The output electrical current *I* (left) and the output electrical power *P*_*E*_ (right) versus the output voltage ${\rm{\Delta }}{V}_{out}$ in a nanochannel. Parameters are the same as those in Fig. 2. (**b**) The energy converting efficiency $\gamma$ (left) and the thermal power ${P}_{th}$ (right) as functions of ${\rm{\Delta }}{V}_{out}$.

The calculation results are plotted in Fig. 6. As expected, the output electrical power ${P}_{E}$ as a function of the voltage ${V}_{out}$ becomes largest when ${V}_{out}={\rm{\Delta }}{V}_{op}/2$. The figure further demonstrates that the energy converting efficiency $\gamma$ also reaches the maximum at ${V}_{out}={\rm{\Delta }}{V}_{op}/2$. It is ascribed to the fact that the thermal power *P*_*th*_ keeps almost invariant with changing ${V}_{out}$ (Data shown with top-right axes of Fig. 6b). Here the physical mechanism is that the total amount of ion flow *Q* is determined by the imposed concentration bias, while the electrical potential bias merely tunes the relative amplitudes of cationic and anionic components. Mathematically, it means the sum of ${Q}_{+}$ and ${Q}_{-}$ relies on $({C}_{max}-{C}_{min})$, while the difference of ${Q}_{+}$ and ${Q}_{-}$ depends on ${V}_{out}$. Hence our analysis indicates that in the real experiments both the absolute and relative energy converting maximums, ${P}_{E}$ and $\gamma$, are attained at half the open-circuit voltage when the load resistance *R*_*L*_ is the same as internal resistance ${R}_{ch}$. And we point out that when we discuss the effect of the load resistance on the power performance, we keep the parameters of nanochannel such as the length of nanochannel and the salt concentration at the two ends invariant, so that the internal resistance and open-circuit voltage are fixed. As we will see, this is not the case when other device parameters are tuned because the ionic distributions vary with the changes of nanochannel parameters, and we have to make strategies to fulfill the demands of maximizing the available power.

#### Optimise output power

Although several types of materials have been explored as the membranes for nanochannel RED, two candidates, the Al_2_O_3_ and SiO_2_, stand out due to their capability of mass production, reliability and low price. The Al_2_O_3_ nanochannel RED have been studied systematically from both experimental and theoretical sides^19,25,40^. Based on 2-dimensional electrokinetic simulation, it has been suggested that positively charged Al_2_O_3_ channels may achieve better energy converting efficiency and output power^40^. The conclusion can be perceived straightforwardly from the inset of Fig. 1 and Fig. 2 of our work: ${\rm{\Delta }}{V}_{\sigma }$ and ${\rm{\Delta }}{V}_{D}$ will point to the same orientation rather than contrarily, once the surface charges on the channel wall are positive; therefore the overall open-circuit voltage is enhanced. Yet from the viewpoint of utilizing the matured silicon process, it is worth exploring SiO_2_-based nanochannel RED optimization as here.

**Figure 7:**
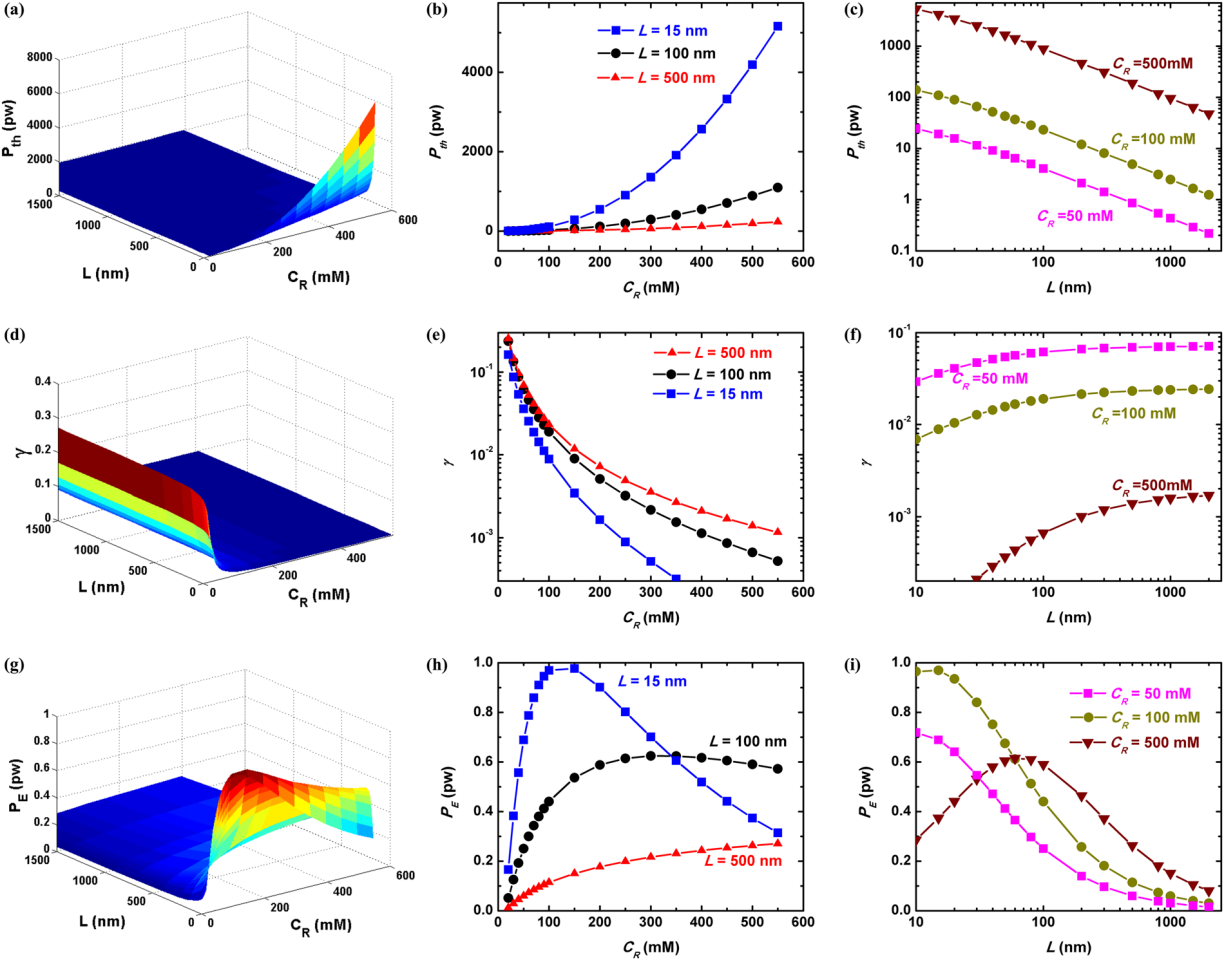
The thermal power ${P}_{th}$, output electrical power *P*_*E*_ and energy converting efficiency $\gamma$ as functions of the imposed NaCl concentration bias ${C}_{R}/{C}_{L}$ and the channel length *L*. Here *C*_*L*_ is fixed at the river situation 10 mM, the channel radius *R* = 5 nm, and the density of surface charges on the channel wall ${\sigma }_{w}=-50$ mM/m^3^. Figures in the left column show 3-dimensional view while those in the middle and right show dependence on *C*_*R*_ and *L* respectively.

The first row of Fig. 7 discusses the dependence of input thermal power ${P}_{th}$ on the NaCl concentration at the sea side *C*_*R*_ and the channel length *L*. ${P}_{th}$ shows monotonous increasing trend with larger *C*_*R*_ while decreasing with *L*. The physical mechanism is straightforwardly illustrated in Eq. 26: both denser NaCl at the sea side and shorter nanochannel would stimulate larger magnitude of diffusion ion flux, which boosts greater thermal power generation.

On the other hand, increasing channel length would foster the energy converting efficiency $\gamma$, while enhancing NaCl concentration at the sea side would do the opposite, as demonstrated by the second row of Fig. 7. From previous discussion, we are aware that $\gamma$ characterizes the ion selectivity of the nanchannel system. Therefore the above results indicate that longer channel and smaller NaCl at the sea side would benefit the ion selectivity. The physical mechanism for the former relation, $\gamma (L)$, is that the longer the channel, the larger the proportion of the imposed NaCl concentration bias $({C}_{max}-{C}_{min})$ would drop within the channel. By quantitatively checking ${C}_{0}(-L\mathrm{/2)}$ and ${C}_{0}(L\mathrm{/2)}$ in Eq. 3, we find that a decreased ion concentration at the diluter end ($z=L/2$) while an increased one at the contrary end ($z=-L/2$) would be resulted in. The physical consequences are that EDL become thicker at ${C}_{min}$ end of the channel while they get thinner at the opposite one, due to the longer nanochannel. In other words, the ion selectivity is reinforced at ${C}_{min}$ end but attenuated at ${C}_{max}$ one, when the nanochannel length increases. Nonetheless, the overall effect is *enhanced* ion selectivity, since the increasing amount of EDL at ${C}_{min}$ end is larger than the decreasing counterpart at the opposite end (note that Debye length ${\lambda }_{D}\propto {C}_{0}^{-\mathrm{1/2}}$, and thus the increase of ${\lambda }_{D}$ at ${C}_{min}$ end is larger than the decrease of ${\lambda }_{D}$ at ${C}_{max}$ one. We plot the normalized NaCl concentration distribution and Debye length along the channel axis in Fig. S1 of the Supplementary Materials for further demonstration).

The above physical picture of ${\lambda }_{D}$-characterized ion selectivity can be also applied to interpret the $\gamma ({C}_{R})$ relation shown in Fig. 7e. The increasing NaCl concentration at the sea side promotes the ion concentration globally inside the channel, and thereby the EDL turn thinner. The attenuated energy converting efficiency is then ascribed to the weakened nanochannel ion selectivity with thinner EDL.

So far we have clarified the physical pictures behind the tuning of input thermal power and energy converting efficiency by nanochannel length and concentration bias. Nonetheless, from the viewpoint of application, it poses several challenges on design as seen in the last row of Fig. 7. First, the output electrical power *P*_*E*_ can not reach the maximum value at either largest or smallest channel length *L*, since it is a product of the input thermal power ${P}_{th}$ and the converting efficiency $\gamma$. By fabricating shorter nanochannels, the input thermal power is enhanced however at the expense of aggravating the energy converting efficiency. On the other hand, by using longer channels, the ion selectivity of the channels is improved and thus the converting efficiency is advanced, while the intensity of diffusion flux becomes attenuated and so the input thermal power declines. Similar dilemma exists for selecting the NaCl concentration at the sea side. The above analysis is summarized in Table 2. Here we remind that a similar trade-off relationship exists for the traditional RED approach by using cation/anion selective membranes^1,2^. By using thinner membrane, the conductance would be improved and thereby the output electrical power is enhanced. However, it is at the expense of attenuating the perm selectivity of the membrane and consequently the energy converting efficiency is reduced. Likewise, weakened water and co-ion permeation by fabricating thicker membrane is beneficial to the energy converting efficiency. Nonetheless, it would be inevitably accompanied by reduced conductivity and therefore, the output electricity would be reduced.

**Table 2:** Dependence of input thermal power ${P}_{th}$ and energy converting efficiency $\gamma$ on the nanochannel length L and the NaCl concentration at denser end ${C}_{max}$.

	*P*_*th*_	γ
*L*	↑⇒↓	↑⇒↑
*C*_*max*_	↑⇒↓	↑⇒↓

In order to address the above challenges, we propose a device design by stepwise usage of the sea/river mixing power as seen in Fig. 1. There are two layers of SiO_2_ membranes segregating the meeting sight between river and sea into three parts. The concentrations of NaCl separated along the flow direction are denoted as ${C}_{max}$ (sea), ${C}_{mid}$ and ${C}_{min}$ (river). According to our calculation shown in Fig. 7, by setting the thickness of first layer near the river side 15 nm and ${C}_{mid}$ 150 mM, the output electrical power reaches the maximum value of ${P}_{E\mathrm{,1}}=0.977\times {10}^{-12}$ W per-nanochannel. It is equivalent to 1.24 kW/m^2^. By further utilizing the NaCl concentration bias ${C}_{mid}/{C}_{max}=150$ mM/600 mM at the second layer of SiO_2_ membrane with $L=15$ nm, another output power density ${P}_{E\mathrm{,2}}\,=$ 92 W/m^2^ is achieved (See Fig. S2 in the Supplementary Materials).

### Recently Emerged ultrathin Nanopores

Recently by fabricating nanopores in single-layer MoS_2_ Membrane, an ultrathin nanofluid device for harvesting the blue energy was demonstrated^23^. The electrical current generated by a KCl concentration bias ${C}_{max}/{C}_{min}\, \sim$ 1 M/1 mM through a 15-nm diameter MoS_2_ nanopore reached several nanoamperes, which implied a power density as huge as 10^6^ W/m^2^ could be gained. Such breakthrough might set a milestone on the road towards blue energy generating, and here we show our theoretical investigation, with particular attention to the properties associated with the atomic thin layers.

**Figure 8:**
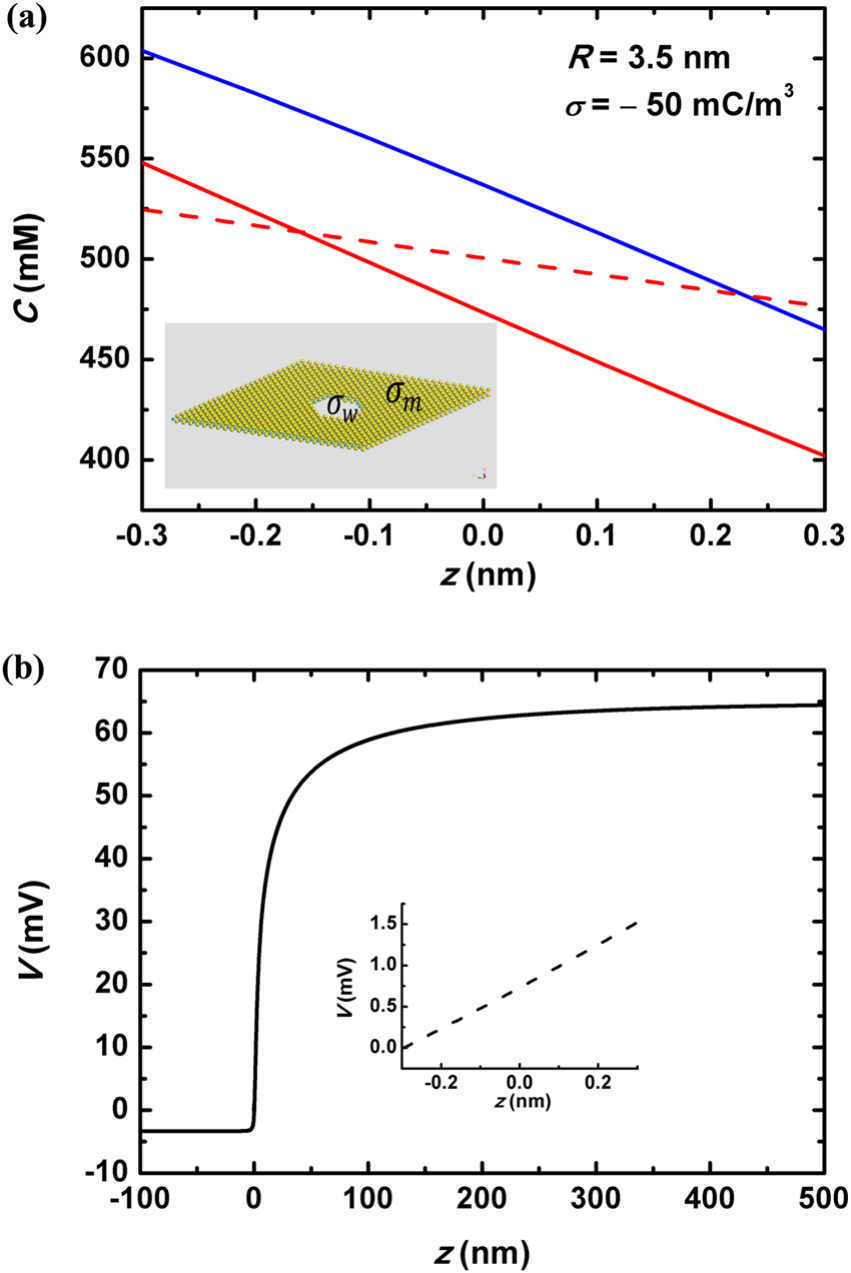
(**a**) Distribution of K^+^ and Cl^−^ concentrations along MoS_2_ nanopore axial direction. Real blue and red lines are *C*_*K*_ and ${C}_{Cl}$ by the 2-D axial-symmetrical model, while dash lines are by space-charge model. *C*_*K*_ and ${C}_{Cl}$ calculated by the latter are fully overlapped inside the pore. The inset is a schematic view of MoS_2_ nanopore. ${\sigma }_{m}$ and ${\sigma }_{w}$ characterize the density of charges on the membrane outside surface and the inner wall surface, respectively. (**b**) The voltage distribution along the pore axial direction under open-circuit state by the 2-D (real line) and space-charge models (inset dash line).

By analyzing the experimental data, our conclusion is that a crucial difference between this ultrathin nanopore system and the previously discussed ones is the role of those charges on the membrane surface. The inset of Fig. 8a shows schematic view of single-layer MoS_2_ nanopore, where the density of charges on the outside surface is labeled by ${\sigma }_{m}$ while that on the inner wall is by ${\sigma }_{w}$. In our previous discussions only the effect of ${\sigma }_{w}$ was considered, while that of ${\sigma }_{m}$ was neglected. The rationale was that the discussed nanochannels were sufficiently long and thus the influence of ${\sigma }_{m}$ on the transport within the channel was trivial. However, this approximation no long stands for the ultrathin MoS_2_ nanopores. This is clearly demonstrated by the conductance saturation behavior under very small KCl concentration as shown in Fig. 2b of ref.^23^. By decreasing the concentration of imposed salt, the major role contributing to the conductance *inside* the nanopore is gradually taken by those induced counterions: ${G}_{pore}\approx {\mu }_{Cl}|{\sigma }_{w}|2\pi R/L$. On the other hand, without considering the ${\sigma }_{m}$-induced charges, the access resistance of the two chambers is ${R}_{acc}\approx {\mathrm{[2}R{C}_{0}e({\mu }_{K}+{\mu }_{Cl})]}^{-1}$. The ultrathin nature of MoS_2_ ($R > L$) leads to the dominance of access resistance over the within-pore one when decreasing the added salt concentration *C*_0_ below a critical value: ${R}_{acc} > 1/{G}_{pore}$. Hence the conductance of the whole nanochannel system is now determined by *R*_*acc*_. It indicates a linear *G*(*C*_0_) relation given very dilute KCl concentration, which contradicts with the experimental reports. The above reduction-to-absurdity suggests that the role of ${\sigma }_{m}$-induced charges are noneligible. Therefore, a 2-dimensional electrokinetic model taking ${\sigma }_{m}$ into account is necessitated.

We establish a 2-dimensional axial-symmetric multi-physical model including Poisson equation for electrostatics and Nernst-Plank equation for ion transport^18,23,25,36^, and perform numerical calculation (Detailed discussion is provided in Section Two-Dimensional Axial-Symmetric Multi-physical Model of the Supplementary Materials). The simulated pore-axial distribution of the cation and anion concentrations is plotted with solid lines in Fig. 8a, where that calculated by the space-charge model is shown with dash lines as comparison. By comparing the solid and dash lines, we find that the space charge model underestimates the amount of concentration drop within the channel. It is ascribed to the effect of ${\sigma }_{m}$ in the above statements. Figure 8b shows the calculated open-circuit voltage distribution along the nanopore axial direction by the 2-D model (real line) and by the space-charge model (dash lines in the inset). We show that by considering ${\sigma }_{m}$, the landscape of saline concentration gradient becomes absolutely different from that by space-charge model. Physically it is attributed to the coupling between nanopore axial and radial transport, and the resulted thermal power is much larger than the estimation by space-charge model. Our quantitative simulation of the open-circuit voltage and short-circuit current by COMSOL then shows better agreement with the experimental measurements.

## Conclusion

We have investigated theoretically the power generation using nanochannel RED. We have illustrated that the competition between the exclusion and diffusion potentials plays the crucial role in dominating the measured current-voltage characteristic, and the existing experimental results using various species of salt, different kinds of materials and channel shapes can be well understood through this analysis. In order to fully utilize the saline concentration bias induced thermal energy, we have proposed a step-wise usage of the RED stimulated by the river and sea meeting in the nanochannels. Our work has offered insights on nanochannel RED based energy harvesting and identified the actual energy accessible for utilization through the river/sea salinity gradient.

## Electronic supplementary material

Supplementary Materials
