# Supplementary material for: Electrokinetic Analysis of Energy Harvest from Natural Salt Gradients in Nanochannels
Source: Sci Rep. 2017 Oct 13;7:13156. doi: 10.1038/s41598-017-13336-w (PMC5640757; doi:10.1038/s41598-017-13336-w)
Supplement: Supplementary file 1 — Supplementary Materials [file 41598_2017_13336_MOESM1_ESM.pdf]

Supplementary Materials for Electrokinetic Analysis of Energy Harvest from Natural Salt Gradients in Nanochannels

Yuhui He[1], Zhuo Huang[1,+], Bowei Chen[1], Makusu Tsutsui[2,*], Xiang Shui Miao[1,*], and Masateru Taniguchi[2]

[1]School of Optical and Electronic Information, Huazhong University of Science and Technology, LuoYu Road, Wuhan 430074, China
[2]The Institute of Scientific and Industrial Research, Osaka University, 8-1 Mihogaoka, Ibaraki, Osaka 567-0047, Japan
[*]corresponding author: tsutsui@sanken.osaka-u.ac.jp and miaoxs@hust.edu.cn
[+]these authors contributed equally to this work

ABSTRACT

The Gibbs free energy released during the mixing of river and sea water has been illustrated as a promising source of clean and renewable energy. Reverse electrodialysis (RED) is one major strategy to gain electrical power from this natural salinity, and recently by utilizing nanochannels a novel mode of this approach has shown improved power density and energy converting efficiency. In this work, we carry out an electrokinetic analysis of the work extracted from RED in the nanochannels. First, we outline the exclusion potential effect induced by the inhomogeneous distribution of extra-counterions along the channel axis. This effect is unique in nanochannel RED and how to optimize it for energy harvesting is the central topic of this work. We then discuss two important indexes of performance, which are the output power density and the energy converting efficiency, and their dependence on the nanochannel parameters such as channel material and geometry. In order to yield maximized output electrical power, we propose a device design by stepwise usage of the saline bias, and the lengths of the nanochannels are optimized to achieve the best trade-off between the input thermal power and the energy converting efficiency.

S1 Comparison with Teorell-Meyer-Sievers Model

By using Eq.4 and 5 and in the main context, we obtain the following expression for current-voltage characteristic based on TMS model:

$$\frac{\partial V_0}{\partial z} = \frac{kT}{e}\frac{D_- - D_+}{(D_- + D_+) + (D_- - D_+)\sin\theta}\frac{\partial \ln C_0}{\partial z} - \frac{\cos\theta}{\pi R^2 C_0 e[(\mu_- + \mu_+) + (\mu_- - \mu_+)\sin\theta]} I_z \tag{S1}$$

where $\theta = \arctan(\sigma_w / eC_0R)$. The $I(V)$ characteristic is then determined by the following relation:

$$V_{out} = \Delta V_{op} - R_{ch} I_z \tag{S2}$$

where

$$R_{ch} = \int_{z=-L/2}^{z=L/2} [\pi R^2 C_0 e[(\mu_- + \mu_+)\sec\theta + (\mu_- - \mu_+)\tan\theta]]^{-1} dz \tag{S3}$$

.

In Fig.2 of the main context, the open-circuit voltage V_0 and E-field along the channel-axial direction E_z calculated by the space-charge model are plotted. Here in Fig.S3a, we further plot that calculated with TMS model by the dash lines. Similarly, in Fig.S3b the $I(V_{out})$ curves calculated by the space-charge model and TMS one are plotted with solid and dash lines respectively.

S2 Comparison with Charge Regulation Model

Quantitatively in charge regulation model, the relation between surface charge density on the channel wall and the imposed saline concentration, $\sigma_w(C_0)$, is calculated in a self-consistent manner. First, a guess value of σ_w is proposed for a given C_0, and then the corresponding surface potential ϕ_s is obtained based on Eq.13 and Eq.14 of the main context[1]. On the other hand, ϕ_s can also be estimated from the Poisson-Boltzmann equation. For example, for cylindrical nanochannel with monovalent ions, it is written as Eq.2 of the main context. By tuning the magnitude of σ_w, the values of ϕ_s calculated by the two approaches get to agree with each other. In this way, we arrive at $\sigma_w(C_0)$ relation as shown in Fig.S5.

However, quantitatively simulated $\sigma_w(C_0)$ by the Charge Regulation Model (solid lines with hollow symbols) does not agree with the fitting values from experiments (dashed lines with solid symbols), as shown in Fig.S5. The former are 1 or 2 orders smaller than the latter, no matter how we tune those parameters of Charge Regulation Model in the reasonable regions. Moreover, the simulated $\sigma_w(C_0)$ by the Charge Regulation Model are 1 or 2 orders smaller than the quantities estimated via other experiments (e.g., Effect of Salt Concentration on the Electrophoretic Speed of a Polyelectrolyte through a Nanopore. Phys. Rev. Lett.2007, 98, 238104). We have to conclude that there should be some deficiency when applying the current Charge regulation model directly to SiO_2 nanopore transport. Yet we are not very clear about the mechanism, and then left further exploring to interested readers.

S3 Two-Dimensional Axial-Symmetric Multi-physical Model

As mentioned in the main context, the MoS_2 nanopore system can be modeled as consisting of a cylindrical nanopore and two chamber. As indicated in Fig.S4a, C_H and C_L are used to designate the bulk concentrations of the concentrated and dilute electrolytes in the two reservoirs, respectively.
The distribution of the electric potential ϕ inside the nanopore is governed by the Poisson equation:

$$\nabla^2\phi = -\frac{\rho_e}{\varepsilon} = \frac{-N_A e(C_+ - C_-)}{\varepsilon_0\varepsilon_r} \tag{S4}$$

Where, ϕ, ρ_e, C_+, C_-, ε_0 and ε_r are the electric potential, net charge density, cation concentration, anion concentration, permittivity of vacuum, dielectric constant of water, respectively. The flux of each ion species consists of the flux distributions from diffusion, electromigration, and convection:

$$\vec{J}_\pm = D_\pm(\mp\frac{C_\pm}{K_B T}\nabla\phi - \nabla C_\pm) + \vec{u}C_\pm \tag{S5}$$

Here, $\vec{J}_+$, $\vec{J}_-$ and $\vec{u}$ are the cation flux, anion flux and velocity vector, respectively. For the steady-state solution, the ion flux satisfies the following continuity equation:

$$\nabla\vec{J}_\pm = 0 \tag{S6}$$

Motion of the incompressible solution is generated by the electric potential and pressure gradient and can be described by the modified Stokes equation and the continuity equation:

$$-\nabla p + \mu\nabla^2\vec{u} + N_A e(C_+ - C_-)\nabla\phi = 0 \qquad \nabla\vec{u} = 0 \tag{S7}$$

Here, p and μ are the pressure and viscosity, respectively. In Eq.S7, the inertial terms in the NavierStokes equation are neglected because the Reynolds number for the solution flow in the nanopore is typically very small. In these equations, the transport of the ion species and the solution are strongly coupled. Finally, the current through the nanopore I_{pore} was calculated by integrating the current density over the cross-sectional area of nanopore as follows:

$$I_{pore} = \int N_A e(\vec{J}_+ - \vec{J}_-)dA \tag{S8}$$

Based on the physical model and referring to the Fig.S4(b), the above equations (Eqs.S4-S8) are solved numerically with the boundary conditions as follows:
Plane CD : $\phi = U, C_\pm = C_H$
Plane EF : $\phi = 0, C_\pm = C_L$
Plane AB and HG : $\frac{\partial\phi}{\partial z} = -\frac{\sigma_m}{\varepsilon_0\varepsilon_r}, \frac{\partial C_\pm}{\partial z} = 0$
Plane AH : $\frac{\partial\phi}{\partial r}|_{r=R} = -\frac{\sigma_w}{\varepsilon_0\varepsilon_r}, \frac{\partial C_\pm}{\partial r}|_{r=R} = 0$
Plane DE : $\frac{\partial\phi}{\partial r}|_{r=0} = 0$

Eq.S4-S8 along with the above boundary conditions were solved by using finite element calculations in COMSOL. Fig.S4(b) shows the typical triangular computation meshes used in the simulation. Since the numerical solution accuracy strongly depends on the mesh size, a refined mesh is necessary in the region near the surface where the dependent variable gradients are pronounced. In this study, finer mesh was applied in the region near the charged surface in order to capture the subtle changes in the ion concentration and electrical potential. Solution independence on the mesh size was carefully studied before reporting the final results. The minimum element spacing inside the nanopore is less than 0.1 nm. The calculation results are confirmed to the grid independent solution by solutions obtained from more dense meshes.

S4 Comparison with Two Extreme Cases

In order to evaluate the power generation performance of real SiO_2 nanochannel, we demonstrate the two extreme cases as comparisons: no perm-selectivity and ideal perm-selectivity. In our ΔV_σ *versus* ΔV_D model, we have demonstrated the open-circuit voltage coming from RED as follows:

$$\frac{\partial V_0}{\partial z} = \frac{k_B T}{e} \frac{D_-\Lambda_- - D_+\Lambda_+}{D_-\Lambda_- + D_+\Lambda_+} \frac{\partial \ln C_0}{\partial z} \tag{S9}$$

For one extreme case: a liquid junction (no EDLs), the surface of the nanochannel is electrically neutral ($\sigma_w = 0$). And the surface will not attract counterions nor repel co-ions, leading to the concentration of cations and anions is equal in the solution. In this case, the open-circuit voltage can be simply obtained owing to $\Lambda_+ = \Lambda_-$:

$$\frac{\partial V_0}{\partial z} = \frac{k_B T}{e} \frac{D_- - D_+}{D_- + D_+} \frac{\partial \ln C_0}{\partial z} \tag{S10}$$

When we directly imposed the sea and river at the two ends of nanochannel, we can calculate

$$V_{open} = -\frac{k_B T}{e} \frac{D_- - D_+}{D_- + D_+} \ln(\frac{C_{max}}{C_{min}}) = 21mV \tag{S11}$$

On the other hand, assuming that SiO_2 nanochannel is the ideal perm-selectivity, there are only cations within nanochannel. The open-circuit voltage can then be obtained by considering $\Lambda_- = 0$:

$$\frac{\partial V_0}{\partial z} = \frac{k_B T}{e} \frac{D_+\Lambda_+}{D_+\Lambda_+} \frac{\partial \ln C_0}{\partial z} \tag{S12}$$

When we directly imposed the sea and river at the two ends of nanochannel, we arrive at

$$V_{open} = \frac{k_B T}{e} \ln(\frac{C_{max}}{C_{min}}) = 103.4mV \tag{S13}$$

From above calculations, we find the open-circuit voltage (21mV and 103.4mV) in two extreme case are both higher than that results in the real SiO_2 nanochannel as shown in Fig.2a of the main context (15mV). The above analysis also shows good agreement with our qualitative anticipation: the orientation of diffusion voltage ΔV_D is from the C_{max} end to the C_{min} end since Cl^- diffuses faster than Na^+, however the direction of exclusion voltage ΔV_σ is from the C_{min} end to the C_{max} end owing to the cation selectivity of SiO_2 nanochannel. The opposite orientations of diffusion voltage ΔV_D and exclusion voltage ΔV_σ make it reasonable that the amplitude of open-circuit voltage in the real SiO_2 nanochannel is smaller than the ideal perm situation. If we hope to change the opposite direction of ΔV_σ *versus* ΔV_D, we can do some chemical modification on SiO_2 surface to reverse ion selectivity. It certainly enhances the power generation performance, but the costs is greater than the performance improvement. Thus we turn our strategy to the designing of nanochannel geometry parameter and salt concentration at two ends of nanochannel in the main context.

References

1. Behrens, S. H. & Grier, D. G. The charge of glass and silica surfaces. *J. Chem. Phys.* **115**, 6716–6721 (2001).

2. Kim, D.-K., Duan, C., Chen, Y.-F. & Majumdar, A. Power generation from concentration gradient by reverse electrodialysis in ion-selective nanochannels. *Microfluidics and Nanofluidics* **9**, 1215–1224 (2010).

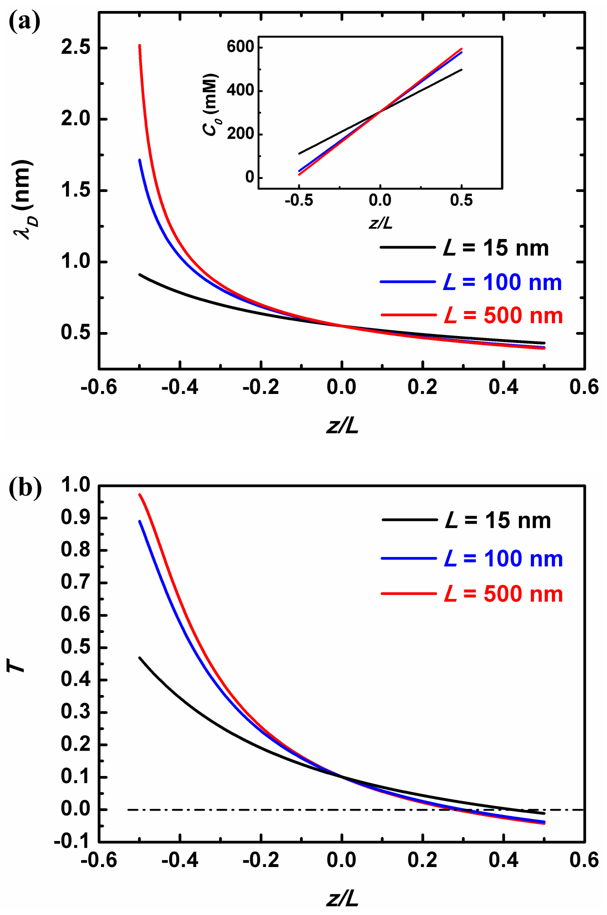

Figure S1. (a) The variation of Debye length λ_D, which characterizes the thickness of EDL, along the channel axis in nanochannels with different channel lengths. Here the imposed salt is NaCl with $C_{max}/C_{min} = 600$ mM/10 mM, the channel radii $R = 5$ nm and $\sigma_w = -50$ mC/m^2. Black, red and blue lines stand for $L = 15$ nm, 100 nm and 500 nm separately. Inset plots the concentration change along the axis $C_0(z/L)$. (b) The landscape of transference number $T = (D_+\Lambda_+ - D_-\Lambda_-)/(D_+\Lambda_+ + D_-\Lambda_-)$, which quantifies the ion selectivity of the nanochannel, along the channel axis.

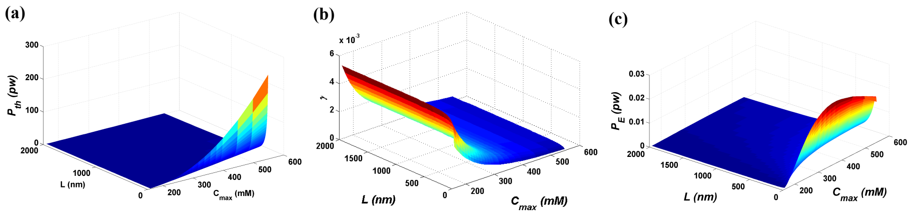

Figure S2. (a) The thermal power P_{th}, (b) the energy converting efficiency γ and (c) output electrical power P_E as functions of the imposed NaCl concentration bias C_{max}/C_{min} and the channel length L. Here C_{min} is fixed at the river situation 150 mM, the channel radius R = 5 nm, and the density of surface charges on the channel wall $\sigma_w = -50$ mM/m^3.

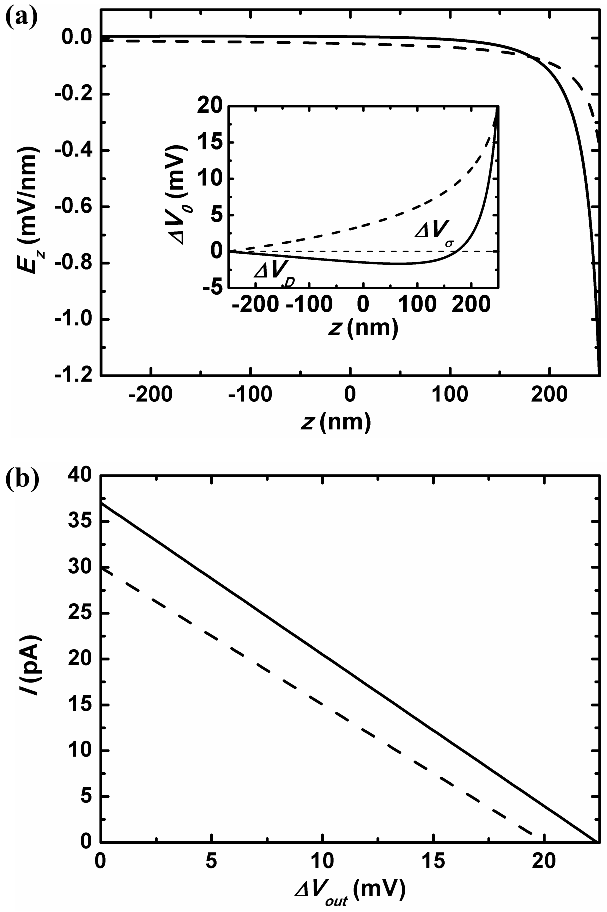

Figure S3. Comparison between space-charge model (solid lines) and TMS one (dash lines). (a) The open-circuit state channel-axial distribution of z-component electrical field $E_z(z)$ in a R=10 nm and L=500 nm nanochannel, where a NaCl concentration bias C_{max}/C_{min} = 600 mM/10 mM is imposed at the two ends and the surface charge density on the wall $\sigma_w = -50$ mC/m^3. Inset plots the corresponding self-built voltage $V_0(z)$. (b) The $I(V_{out})$ curves simulated by space-charge model (real line) and by TMS (dash line).

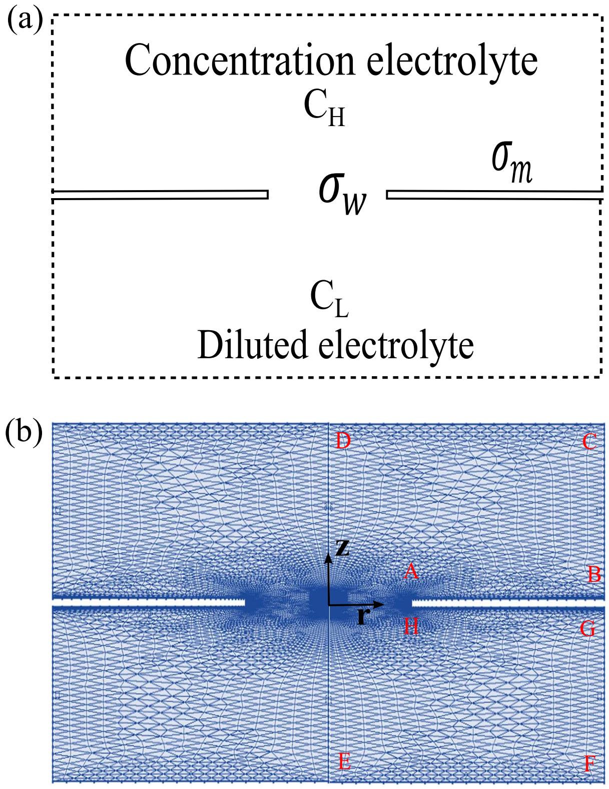

Figure S4. 2-dimensional axial-symmetric multi-physical model (a) Physical model of the MoS_2 nanopore system. (b) Typical computation mesh used in the numerical simulation.

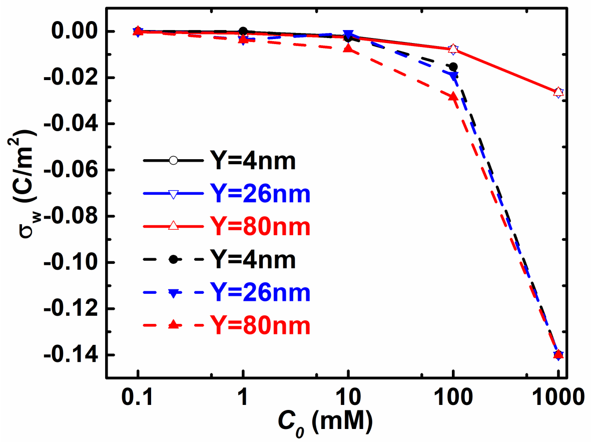

Figure S5. The calculated density of surface charges on the channel wall σ_w as a function of the imposed KCl concentration C_0, by fitting the above experiments[2] (shown in dashed lines with solid symbols) and by Charge Regulation Model (shown in lines with hollow symbols).
